# Literary and Scientific

**Published:** 1869-12

**Authors:** 


### Literary and Scientific.

*Man in Genesis and in Geology;* or, The Bible Account of Man's Creation Tested by Scientific Theories of his Origin and Antiquity. By Joseph P. Thompson, D.D., LL.D. 1 vol. 12mo. Price $1.

To reconcile Revelation with Science; to go back through Ethnology to the origin of the human race; and to examine critically the theories of the philosophers and writers on the Development Theory, in order to arrive at the facts, and thus settle the question, is the object of the author. Rev. Dr. Thompson, of the Broadway Tabernacle Church, in New York, is considered one of the ripest scholars in America. The book contains the gist of a vast amount of Scriptural and scientific knowledge, reviewing all authorities to date. Published by S. R. Wells, New York.

*A Philosophy of Heaven, Earth and the Millenium.* "And God called the firmament Heaven."—Gen. i. 8. By JAMES A. SPURLOCK, a Member of the Missouri Bar. W. J. Gilbert, Publishers, St. Louis, Mo.

*Notes in England and Italy.* By Mrs. HAWTHORNE. New York: G. P. Putnam & Son. 1869.

THOSE of our friends intending the "grand tour," will find many suggestive "art notes," and hints by which both their eyes and footsteps may be profitably guided, after crossing the great waters. The observant author somewhat checks our own desire of visiting the old world by one of her descriptions:

"We, of the other side of the Atlantic, have not the remotest idea how dirty a person can be who has not been washed for nearly three thousand years. * * * It is only in Europe that one can see a dirty face, and it is necessary to come to Europe to comprehend it. Description will not avail."

Fortunately, her graphic pictures of old world art, letters, antiquities, etc., redeem the prospect thus ominously shaded.

*Through Night to Light.* A Novel. By FREDERICH SPIELHAGEN. Author's Edition. New York: Leypoldt & Holt. 1870.

LESS known perhaps in this country than *Freytag* or *Auerbach*, Spielhagen surpasses both in strength, vigor, and depth of passion and thought. Prof. Schele de Vere is the translator into excellent English, and it is for sale by Keen & Cooke.

*The Annals of Iowa.* Published Quarterly by the State Historical Society at Iowa City. October, 1869. Edited by Sanford W. Huff, M.D., Corresponding Secretary.

WE find in this number some interesting details on the Materia Medica and practice of the Indian tribes of the West, which we extract:

